# Can a disease-specific education program augment self-management skills and improve Health-Related Quality of Life in people with hip or knee osteoarthritis?

**DOI:** 10.1186/1471-2474-7-90

**Published:** 2006-11-30

**Authors:** Richard H Osborne, Rachelle Buchbinder, Ilana N Ackerman

**Affiliations:** 1 Centre for Rheumatic Diseases, Royal Melbourne Hospital and The University of Melbourne, Melbourne, Australia; 2 Monash Department of Clinical Epidemiology at Cabrini Hospital and Department of Epidemiology and Preventive Medicine, Monash University, Malvern, Australia

## Abstract

**Background:**

Patient education and self-management programs are offered in many countries to people with chronic conditions such as osteoarthritis (OA). The most well-known is the disease-specific Stanford Arthritis Self-Management Program (ASMP). While Australian and international clinical guidelines promote the concept of self-management for OA, there is currently little evidence to support the use of the ASMP. Several meta-analyses have reported that arthritis self-management programs had minimal or no effect on reducing pain and disability. However, previous studies have had methodological shortcomings including the use of outcome measures which do not accurately reflect program goals. Additionally, limited cost-effectiveness analyses have been undertaken and the cost-utility of the program has not been explored.

**Methods/design:**

This study is a randomised controlled trial to determine the efficacy (in terms of Health-Related Quality of Life and self-management skills) and cost-utility of a 6-week group-based Stanford ASMP for people with hip or knee OA.

Six hundred participants referred to an orthopaedic surgeon or rheumatologist for hip or knee OA will be recruited from outpatient clinics at 2 public hospitals and community-based private practices within 2 private hospital settings in Victoria, Australia. Participants must be 18 years or over, fluent in English and able to attend ASMP sessions. Exclusion criteria include cognitive dysfunction, previous participation in self-management programs and placement on a waiting list for joint replacement surgery or scheduled joint replacement.

Eligible, consenting participants will be randomised to an intervention group (who receive the ASMP and an arthritis self-management book) or a control group (who receive the book only). Follow-up will be at 6 weeks, 3 months and 12 months using standardised self-report measures. The primary outcome is Health-Related Quality of Life at 12 months, measured using the Assessment of Quality of Life instrument. Secondary outcome measures include the Health Education Impact Questionnaire, Western Ontario and McMaster Universities Osteoarthritis Index (pain subscale and total scores), Kessler Psychological Distress Scale and the Hip and Knee Multi-Attribute Priority Tool. Cost-utility analyses will be undertaken using administrative records and self-report data. A subgroup of 100 participants will undergo qualitative interviews to explore the broader potential impacts of the ASMP.

**Discussion:**

Using an innovative design combining both quantitative and qualitative components, this project will provide high quality data to facilitate evidence-based recommendations regarding the ASMP.

## Background

Osteoarthritis (OA) is a common chronic disease that poses particular challenges to patients and health professionals as there is currently no cure for the condition. Consequently, the majority of chronic disease care and management is undertaken by the individual who may only have brief and infrequent interactions with the health care system. Effective management of OA therefore requires *self-management *of the condition. Patient education programs aim to impart knowledge and skills to individuals so that they may better manage their arthritis. A widely-recognised program is the Arthritis Self-Management Program (ASMP), developed at the Stanford University Medical Centre in the early 1980s by Lorig and colleagues [1]. The program addresses pain management, healthy behaviours, communication with doctors and disease-specific information [2]. An important aspect of the program is the bringing together of individuals in a similar situation for mutual support and to foster a sense of self-efficacy [3,4]. Self-efficacy is based on social cognitive theory [5] and relates to an individual's confidence and ability to perform healthy behaviours.

Osborne et al [6] described the core concepts promoted in patient self-management as:

• engagement in activities which promote health, build physiological reserve, and prevent adverse sequelae;

• appropriate interaction with healthcare providers and adherence to recommended treatments;

• monitoring of physical and emotional status and making appropriate management decisions on the basis of the results of self-monitoring; and

• management of the effects of illness on an individual's emotions, self-esteem, relationships with others and ability to function in important roles.

A key criticism of previous studies evaluating the ASMP is the use of outcome measures which do not accurately reflect the goals of the programs. Table 1 presents the pooled effect sizes from recent meta-analyses of studies evaluating self-management education programs (not restricted to the Stanford ASMP). Earlier studies have focussed on measures of pain and disability [7-9], which overall, have remained unchanged or only slightly improved after self-management programs. A small beneficial effect of self-management on psychological outcomes was reported [10]; although only 3 studies used the Stanford ASMP model and variability in the individual effect sizes (range -0.19 to 0.33) means that firm conclusions cannot be drawn. Most recently, a large study conducted in the United Kingdom reported significant improvements in anxiety and self-efficacy following an arthritis self-management program, although the mean gains were small and unlikely to be clinically important [11].

As non-specific chronic disease self-management programs have been found to be beneficial for other patient groups in terms of reduced blood pressure, fewer asthma attacks and lower blood glucose levels [8,9], it is likely that the outcomes of the ASMP are yet to be appropriately and comprehensively assessed. This is supported by Newman et al [12], who considered that the ASMP was primarily directed towards strategies to cope with symptoms and their sequelae, rather than the reduction of pain and disability. Thus, the ASMP focuses on changing an individual's perceptions of pain and the development of strategies to manage the disabling effects of arthritis. The intended impact of the program would therefore be better described as improving an individual's self-management skills set, enhancing their knowledge of arthritis and appropriate use of healthcare and further down the causal chain, improving their Health-Related Quality of Life (HRQoL). These constructs have been poorly-measured to date but could provide valuable information about the potential broader impacts of the ASMP.

Another limitation of earlier studies investigating the ASMP has been the use of unrepresentative samples. Much of the initial research was undertaken by the developers of the ASMP using randomised convenience samples recruited from the community [1,13]. Such samples are unlikely to be representative of the wider population with OA, particularly with respect to the level of education attained. For example, the largest study included 695 individuals where 73% of the sample had more than 12 years of education and of these, 29% had more than 16 years of education [13]. Previous studies have used heterogeneous study samples which have included people with OA, rheumatoid arthritis and fibromyalgia [7]. Further research involving representative and well-characterised samples is required to improve the generalisability of the results.

Although current Australian and international clinical guidelines promote the concept of self-management for osteoarthritis [14,15], there is presently little evidence to support the ASMP. This lack of evidence may be a barrier to clinicians referring patients to the program. Our earlier research has shown that ASMPs are under-utilised by people with arthritis and that only a small proportion of self-management referrals come from health professionals [6,16]. While the ASMP has been applied in some community settings for many years, its overall impact on the wellbeing of people with OA remains unknown. The present study will provide much-needed data on the efficacy of the ASMP with respect to HRQoL and self-management skills sets; it will also evaluate the cost-utility and applicability of this intervention for people with lower limb OA.

## Methods/design

### Aim

The primary aim of this study is to determine the efficacy of a 6-week, group-based ASMP on HRQoL and self-management skills in people with hip or knee OA.

### Study design

This study is a randomised controlled trial with a 12 month follow-up period. Although the blinding of participants would be ideal, we did not consider this feasible as the possibility of attending a 6 week program needs to be explained to potential participants in order to ascertain their willingness to take part in the study.

**Figure 1:**
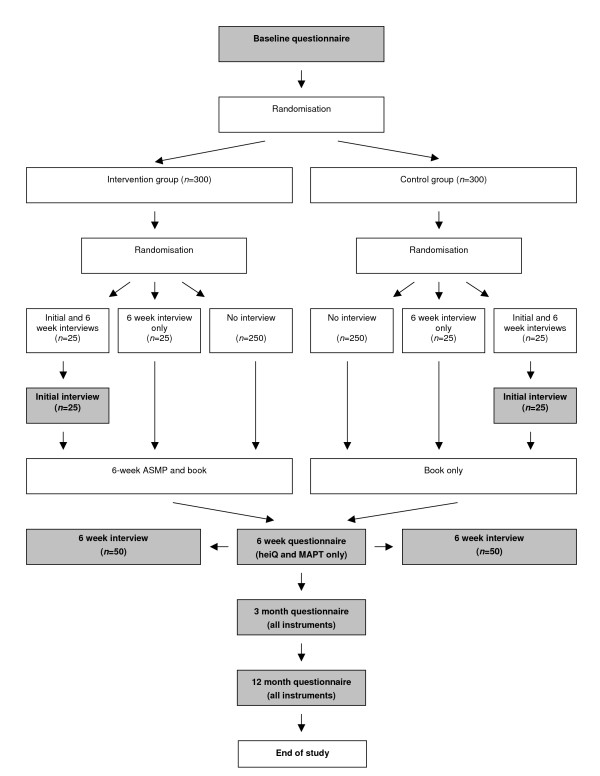
Study design and assessment points.

**Figure 2:**
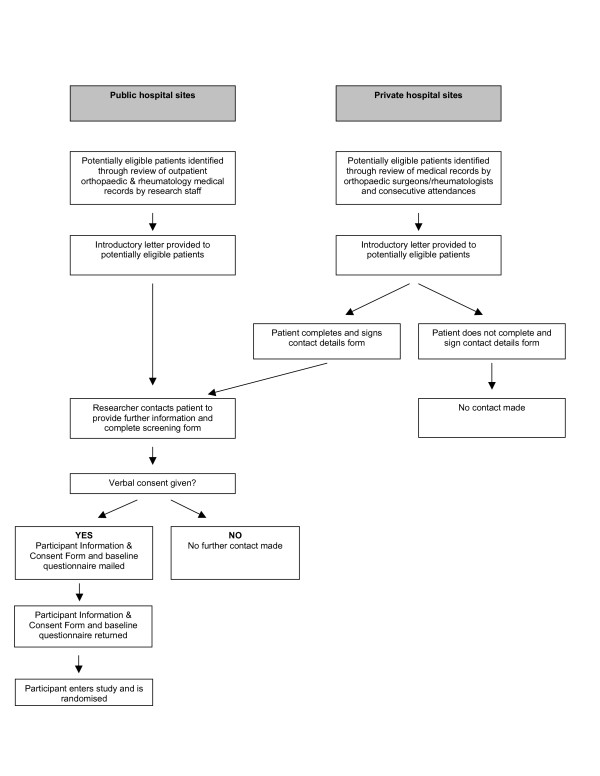
Identification and recruitment of study participants.

In the present study, participants will be allocated to an intervention group who will undergo the Stanford ASMP and receive an arthritis self-management book [2] or to a control group who will receive the book only. The study has both quantitative and qualitative components; an overview of the recruitment method, randomisation process and follow-up procedures is provided in Figures 1 and 2.

### Ethics

Ethics approval has been received from the Human Research Ethics Committees of Barwon Health, Cabrini Hospital, Epworth Hospital and The University of Melbourne.

### Eligibility criteria

The eligibility criteria for this study are listed in Table 2. Determination of eligibility will involve a two-step process commencing with review of the potential participant's medical record and subsequent telephone screening. A diagnosis of hip or knee OA will be extracted from radiology reports; where this information is not stated explicitly, medical records and radiographs will be used to diagnose OA according to American College of Rheumatology criteria [17,18]

### Identification and recruitment of study participants

Six hundred participants referred to an orthopaedic surgeon or rheumatologist for hip or knee OA will be recruited from outpatient clinics at 2 public hospitals and community-based private practices located within 2 private hospital settings in Victoria, Australia. Potentially eligible individuals will be identified by both retrospective review of recent medical records and prospective identification of consecutive presentations attending orthopaedic surgeons and rheumatologists associated with the study hospitals. Due to differences in the structure of public and private health care systems in Australia, the recruitment procedure at each of the sites will be slightly different. At the private practices, identification of potentially eligible participants will be undertaken by the treating specialist and a letter inviting participation will be provided. Written consent will be required before contact can be made by the researchers. At the public hospital sites, potentially eligible participants will be sent an information letter and then contacted by a researcher. The recruitment and screening process is summarised in Figure 2.

A researcher will then telephone potentially eligible participants to provide detailed information about the study. At this time, a screening form will be completed to verify that the individual meets the eligibility criteria. Potential participants will be asked about their preferences for ASMPs with respect to scheduling and the preferred format of the program (eg 6-week course or provision of a book). Individuals who do not provide verbal consent will be asked to list up to three reasons for not participating in the study. This information will be used to estimate the feasibility of conducting the ASMP in a 'real world' setting and for translating the program into clinical practice.

People who meet the specified eligibility criteria and provide verbal consent will then be sent a consent form and baseline questionnaire.

### Randomisation

Once a completed consent form and baseline questionnaire have been returned to the researchers, the participant is considered to have entered the study and will be randomised to either the intervention or control groups, stratified by study site. For each site, a computer-generated random numbers table will be used to assign group allocation according to permuted blocks of 4 or 6. A second randomisation procedure, stratified by treatment group, will be used to select a subgroup of 100 participants who will undergo up to 2 qualitative telephone interviews (conducted at baseline and 6 weeks, or 6 weeks only).

Group allocation will be concealed using opaque sealed envelopes. Upon receipt of a completed consent form and baseline questionnaire, the group allocation envelope will be opened at the study co-ordinating centre (Centre for Rheumatic Diseases, The University of Melbourne) by a research assistant not associated with this study. To further ensure the integrity of the treatment allocation, this process will be observed by a second staff member and recorded with a signature and date. At this point, participants will be informed of their group allocation.

### Intervention

The active intervention group will take part in the Stanford ASMP. The program consists of a weekly 2.5 hour session for 6 weeks. The program will be held in a variety of locations in metropolitan Melbourne and Geelong and at a variety of times (day, evening, weekday and weekend) to maximise attendance. Each course will have up to 12 participants. The course is delivered in a prescriptive format by two leaders and covers the following key areas [6]:

• management of pain and fatigue

• physical activity

• medication usage

• managing anger, fear and frustration

• solving health-related problems

• communication with doctors

For this study, one peer leader (who has arthritis or personal experience of arthritis) and one health professional leader (for example, a nurse or physiotherapist) will be used. This model has worked well in our pilot research and anecdotal reports indicate that this method may be preferred by referring clinicians. Course leaders who have undergone training in the Stanford ASMP model will be recruited and additional leaders (where required) will be trained according to the Stanford course leader protocol. Regular course leader meetings will be held to ensure standardised leader quality and high retention rates.

Participants will also receive an arthritis self-management book [2]. Although the primary component of the ASMP is group-based education and close interaction with 'like' individuals, the course also encourages directed home-based learning and practice of skills learned in the sessions. These activities are supported by the arthritis self-management book.

### Control

The control group will not receive the ASMP but will be sent an arthritis self-management book [2]. There is currently no evidence to suggest that provision of this book alone is beneficial, although to date, this has only been assessed in one study which investigated the effect of the ASMP for people with OA, rheumatoid arthritis or fibromyalgia [19]. After 4 months, no change in pain, self-efficacy, disability or mental health was observed for the control group which received the Arthritis Self-Help book only.

### Outcome measures

Participants will be assessed using a range of standardised, self-report measures which include:

#### 1. The Assessment of Quality of Life (AQoL) instrument

The primary outcome for this RCT is HRQoL at 12 months, as measured by the AQoL instrument. This outcome was chosen to reflect the potential longer-term benefits of the multi-faceted ASMP [6]. The AQoL instrument is an Australian generic utility tool which can be used for health economic evaluation through estimation of utilities and calculation of Quality-Adjusted Life Years (QALYs). The four dimensions covered by this instrument include Independent Living, Social Relationships, Physical Senses and Psychological Wellbeing. The AQoL instrument has strong psychometric properties and is more sensitive and responsive than many other widely-used scales including the Medical Outcomes Study 36-Item Short Form (SF-36) [20,21]. The AQoL utility score ranges from -0.04 (worst possible HRQoL) to 1.00 (full HRQoL). A minimal important difference for the AQoL instrument is considered to be between 0.03 and 0.08 AQoL units [22]. For the present study, an increase of 0.05 AQoL units will be regarded as a clinically important improvement in HRQoL.

#### 2. The Health Education Impact Questionnaire (heiQ)

The heiQ was developed to evaluate the intended effects of self-management programs and was found to be reliable and responsive to change in an Australian validation study [6,23]. It has since been administered to over 1300 Australians with chronic conditions who have attended self-management programs [24]. The heiQ contains 42 questions and covers 8 dimensions which include: Positive and active engagement in life, Health behaviour change, Skill and technique acquisition, Constructive attitudes and approaches, Self monitoring and insight, Health service navigation, Social integration and Emotional wellbeing. Each dimension produces an overall score from 1 (strongly disagree) to 6 (strongly agree).

#### 3. The Western Ontario and McMaster Universities OA (WOMAC) Index

The WOMAC Index is a disease-specific measure of health status and is widely used in OA research. The validity, reliability and responsiveness of this measure have been demonstrated in an extensive range of studies [25]. The WOMAC Index consists of 24 questions which cover pain, stiffness and fatigue and produces a total score which can be transformed to a 0 (best possible health) to 100 (worst possible health) scale. The pain subscale score will also be reported.

#### 4. The Kessler Psychological Distress Scale (K10)

The K10 instrument provides a measure of psychological distress [26] and is used in the World Health Organisation World Mental Health Survey and Australian population health surveys. High K10 scores representing high distress are strong predictors of affective disorders such as depression and anxiety [27]. The scale has demonstrated discriminant validity in United States National Health Interview Surveys [26]. The K10 consists of 10 questions about anxiety, depression and worry and produces a total score ranging from 10 (lowest psychological distress) to 50 (highest psychological distress).

#### 5. The Hip and Knee Multi-Attribute Priority Tool (MAPT)

The MAPT was designed as a measure of arthritis disease severity [28]. The MAPT was developed through extensive consultation with patients, orthopaedic surgeons and other health professionals. The MAPT contains 11 items which cover areas such as self-care, enjoyment of life, the economic impact of arthritis and deterioration. A validation study involving over 900 people with hip or knee arthritis demonstrated construct validity, good test-retest reliability and responsiveness to change [28]. The MAPT produces a score from 0 (least disease severity) to 100 (greatest disease severity).

#### 6. Health services use

The (self-reported) number of visits to health professionals during the previous month will be collected from all participants. The costs of consulting health professionals will be calculated using published prices for medical and allied health costs. Additionally, if specific consent is provided, Australian Health Insurance Commission data will also be obtained regarding the use and cost of prescription medications, medical consultations, pathology tests and hospitalisations during the study period.

#### 7. Community services use

Information about the use of both paid and unpaid community assistance (for example, home help and Meals on Wheels) will also be collected by postal survey.

### Follow-up procedures

Participants will receive follow-up questionnaires at 6 weeks (heiQ and MAPT instruments only), 3 months (full range of instruments) and 12 months (full range of instruments). Reply-paid envelopes will be provided to maximise response rates. At each follow-up assessment, participants will be asked whether they have attended an Arthritis Self-Management Program since entering the study to detect potential cross-over from the control group.

### Qualitative component

Recent research suggests that questionnaire-based outcome assessment may not accurately reflect people's experiences of chronic disease self-management [29,30]. Given these findings, this study will incorporate an additional qualitative component to corroborate the questionnaire-based data collected and to elicit detailed information about participants' expectations of the ASMP and perceived outcomes following the program. A subgroup of 100 participants will be selected for the qualitative component. A Solomon four-group design will be used to minimise the potential impact of behavioural changes prompted by the interviews [31,32]. This design incorporates two groups of participants who do not undergo a baseline interview, allowing both the potential effect of the intervention *and *the potential effect of the interview to be evaluated. As illustrated in Figure 1, 50 participants will undergo both baseline and 6 week interviews (25 participants from the intervention group and 25 participants from the control group). A further 50 participants (25 from the intervention group and 25 from the control group) will undergo interviews at 6 weeks only. The semi-structured telephone interviews (approximately 1 hour in duration) will be conducted by two researchers trained in qualitative interview techniques.

The qualitative interviews may also reveal the presence of 'response shift', a potential change in perspective as a result of attending the ASMP [30,33]. Response shift has been purported to confound the traditional pre-post assessment methods used to evaluate the outcomes of health care interventions. In the context of self-management education programs, response shift may arise when a person re-evaluates themselves after a program (retrospectively) as being better or worse *before *the course than they had thought. Response shift can be categorised as negative (when a person realises they were worse before the course than they had first thought) or positive (when a person realises they were better before the course than they had first thought). In a study of 39 people who completed an arthritis or chronic disease self-management program, Osborne et al [30] found that 31% experienced a negative response shift, while 20% experienced a positive response shift. The effect of this change in perspective on outcomes such as HRQoL has not yet been explored and will be evaluated in this study by comparing qualitative and quantitative data.

### Sample size considerations

The sample size calculations for this study are based on the primary outcome measure (AQoL instrument). Our pilot research involving people with OA (*n *= 39) showed a mean (SD) improvement in the 6-month AQoL score of 0.04 (0.21) following the ASMP. Given that some of this sample had end-stage joint disease for which conservative management was unlikely to be effective, we expect the effect of the ASMP to be larger than this in people with less severe OA. It is also important to note that the proportion of participants who reported a substantial benefit (an increase in AQoL score of greater than 0.05 utility units) was about 43%, whereas substantial decline occurred in 20% of participants. Using the proportion of people who report an improvement of more than 0.05 AQoL utility units as an outcome, a sample size of 600 (300 participants in each group) will provide sufficient power to detect an additional 10% of participants in the intervention group reporting a minimal important improvement (assuming 30% of control group who report improvement, beta = 0.80, alpha = 0.05, two-tailed).

No data are available on the proportion of people who might experience a clinically important improvement in HRQoL over the study period without receiving the active intervention, although earlier RCTs conducted by the developers of the ASMP have found minimal improvement for no-treatment control groups for up to 1 year (average improvement <2% for pain and <1% for depression) [34,35]. Based on the findings of Solomon et al [19], provision of the arthritis self-management book to the control group in the present study is anticipated to have a negligible impact. However, given the labile nature of OA, it is possible that the control group may experience fluctuations in wellbeing over the study period. Therefore, a 10% advantage for the intervention group is considered to be a conservative estimate of benefit.

### Planned statistical and qualitative analyses

An intention-to-treat analysis will be performed using all randomised participants who provide any post-baseline data [36].

The intervention and control groups will be examined for baseline comparability with respect to demographic and other factors. Analysis of covariance will be used to assess differences in 12 month outcomes between groups after adjusting for baseline differences. Repeated measures analysis using linear mixed models will assess the constancy of any effects of the ASMP over time.

A cost-utility analysis will be undertaken as part of this research. Standard economic evaluation for clinical trials will be used to assess differences in resource use and health outcomes between groups [37]. The incremental cost or savings per extra person with a clinically significant improvement per Quality-Adjusted Life Year (QALY) compared to the control group will also be calculated. A clinically significant improvement in HRQoL will be regarded as a utility change of at least 0.05 AQoL units [22].

Qualitative analysis techniques will be used for data obtained from the telephone interviews. Interview tapes will be transcribed for analysis; coding of transcripts and any supplementary written notes will be performed using codes developed from the data. Data analysis will be ongoing and will run parallel to the data collection with the objective being to transcribe data from the interview before moving onto the next interview (where feasible). Thematic analysis from the transcripts will be used and results will be discussed according to themes identified from the data.

### Time frame

This study has a 3 year time frame. It is anticipated that identification of potential study participants and recruitment will commence in October 2006. Follow-up assessment will continue until July 2008, after which time data analysis will be performed and a final report will be drafted.

## Discussion

In Australia, the ASMP is highly endorsed by Arthritis Foundations and government agencies, yet the evidence base to justify this support is very weak. International clinical guidelines together with United States and Australian National Arthritis Action Plans provide clear statements that people with OA should be referred to self-management courses as part of routine clinical practice. However, good quality data from OA-specific clinical trials are not available to justify these recommendations.

This project will provide high quality data to facilitate evidence-based recommendations regarding the ASMP. The study population is well-defined and likely to be representative of the target group who might be expected to benefit from the ASMP. The course leaders will be highly trained and the course quality will be tightly controlled. Finally, the outcome measures to be used in this study were selected to encompass key data for a wide range of stakeholders including consumers, clinicians, policymakers and funding bodies.

If the study demonstrates that the ASMP is effective, this will support referral to the program by clinicians caring for people with OA. Endorsement of the ASMP by medical professionals could result in a relatively inexpensive modality being made more readily available to the millions of Australians suffering from OA. Furthermore, if this research finds the ASMP to be effective, it will provide governmental agencies and non-government organisations with evidence to facilitate the appropriate dissemination of this program. In contrast, if the program is not found to be effective then the resources spent in this area could be re-directed to other evidence-based interventions.

## Competing interests

The author(s) declare that they have no competing interests.

## Authors' contributions

RO and RB were responsible for writing the study protocol, with additional detail provided by IA. IA and RO drafted this manuscript and RB provided comments on the drafts. All authors have approved the final version.

## Pre-publication history

The pre-publication history for this paper can be accessed here:



## Figures and Tables

**Table 1:** Pooled effect sizes from published meta-analyses evaluating self-management programs for arthritis

Outcome	Meta-analysis	Number of studies	Pooled effect size (95%CI)	Interpretation
Pain	Warsi et al 2003 [7]	16	0.12 (0.00 to 0.24)	No effect
	Warsi et al 2004 [8]	16	0.12 (0.00 to 0.24)	No effect
	Chodosh et al 2005 [9]	14	-0.06 (-0.10 to -0.02)	Small effect, favours intervention group
Disability	Warsi et al 2003 [7]	12	0.07 (0.00 to 0.15)	No effect
	Warsi et al 2004 [8]	12	0.07 (0.00 to 0.15)	No effect
	Chodosh et al 2005 [9]	12	-0.06 (-0.10 to -0.02)	Small effect, favours intervention group
Physical outcomes*	Devos-Comby et al 2006 [10]	12	0.09 (-0.01 to 0.19)	No effect
Psychological outcomes^†^	Devos-Comby et al 2006 [10]	9	0.20 (0.08 to 0.33)	Small effect, favours intervention group
Impairment^#^	Devos-Comby et al 2006 [10]	3	0.04 (-0.25 to 0.34)	No effect

*Includes measures of pain, disability, physical functioning, arthritis impact and mobility [10]

^†^Includes measures of psychological disability, mental functioning, self-efficacy and depressive symptoms [10]

^#^Includes physiological measures and performance tests [10]

**Table 2:** Eligibility criteria

Inclusion criteria	Exclusion criteria
• aged 18 years or over	• cognitive dysfunction
• diagnosis of hip or knee OA*	• previous participation in an ASMP or similar program
• referred to an orthopaedic surgeon or rheumatologist for hip or knee OA	• placement on an orthopaedic waiting list for joint replacement surgery or scheduled joint replacement surgery
• sufficient English language skills to self-complete written questionnaires	
• a reasonable expectation that all 6 sessions of the ASMP could be attended if randomised to the intervention group	

*A diagnosis of hip or knee OA will be extracted from radiology reports; where this information is not stated explicitly, medical records and radiographs will be used to diagnose OA according to American College of Rheumatology criteria [17, 18]
